# Eosinophilic Granulomatosis With Polyangiitis Presenting as Mononeuritis Multiplex in a Diabetic Patient: Diagnostic and Therapeutic Challenges

**DOI:** 10.7759/cureus.98219

**Published:** 2025-12-01

**Authors:** Waqas Ahmed, Muhammad Zeeshan Khan, Rameen Saeed, Kamran Nazir, Muhammad Farooq

**Affiliations:** 1 Respiratory Medicine, Lancashire Teaching Hospital, Chorley, GBR; 2 Acute Medicine, Aintree University Hospital, Liverpool, GBR; 3 Internal Medicine, Farooq Hospital, Lahore, PAK; 4 Medicine, Lancashire Teaching Hospital, Chorley, GBR; 5 Cardiology, Lancashire Teaching Hospitals NHS Foundation Trust, Preston, GBR

**Keywords:** diabetic peripheral neuropathy (dpn), drop-foot, eosinophilic granulomatosis with polyangiitis (egpa), plasmapheresis treatment, pulmonary hemorrhage

## Abstract

Eosinophilic granulomatosis with polyangiitis (EGPA) is a rare antineutrophil cytoplasmic antibody (ANCA)-associated small-vessel vasculitis that may present with asthma, eosinophilia, pulmonary involvement, and peripheral neuropathy. Distinguishing EGPA-related vasculitic neuropathy from diabetic neuropathy can be particularly challenging in patients with long-standing diabetes mellitus. We present a case of a 64-year-old man with type 2 diabetes, chronic kidney disease, and asthma, who developed systemic symptoms, mononeuritis multiplex, and pulmonary haemorrhage. Laboratory tests revealed marked eosinophilia and strongly positive myeloperoxidase (MPO)-ANCA, while electrophysiological studies demonstrated asymmetric axonal neuropathy. A multidisciplinary assessment by neurology and rheumatology confirmed the diagnosis of EGPA with vasculitic neuropathy, following which treatment with corticosteroids, cyclophosphamide, plasmapheresis, and rituximab resulted in significant clinical improvement. This case highlights the importance of recognizing atypical or asymmetric neuropathies in diabetic patients and considering EGPA early to prevent irreversible organ damage.

## Introduction

Eosinophilic granulomatosis with polyangiitis (EGPA), formerly Churg-Strauss syndrome, is a rare antineutrophil cytoplasmic antibody (ANCA)-associated small-vessel vasculitis that causes eosinophilic tissue infiltration and extravascular granulomas and can affect multiple organ systems, most commonly the lungs and peripheral nerves and, less frequently, the kidneys and heart [1,2]. EGPA typically progresses through three overlapping phases: a prodromal phase with asthma and allergic rhinitis, an eosinophilic phase characterized by tissue infiltration, and a vasculitic phase involving systemic small vessels [3]. Peripheral neuropathy occurs in up to 75% of patients, most commonly manifesting as painful, asymmetric mononeuritis multiplex [4]. Diagnosing EGPA in patients with diabetes mellitus presents a particular challenge. Diabetic neuropathy typically presents as a distal symmetric polyneuropathy, whereas vasculitic neuropathy is asymmetric, patchy, and may be accompanied by systemic features [5-7]. Electrophysiological studies, serologic testing (myeloperoxidase (MPO)-ANCA/perinuclear antineutrophil cytoplasmic antibodies (p-ANCA)) [6], and careful clinical evaluation are critical for accurate differentiation. Early recognition is essential to initiate immunosuppressive therapy like rituximab and prevent irreversible organ damage [8].

## Case presentation

A 64-year-old man with type 2 diabetes mellitus, stage 3 chronic kidney disease, and asthma initially presented to his general practitioner with persistent fever, myalgia, and lethargy following a recent travel to South Asia. He was started on empirical antibiotics for a presumed bacterial infection, but his symptoms did not improve. Shortly afterwards, he was admitted with chest pain and treated for acute coronary syndrome. During this hospitalization, his clinical condition evolved in a way that became increasingly dominated by neurological symptoms.

While in the hospital, the patient began to develop subtle distal sensory symptoms, initially described as intermittent numbness in his left hand and foot. These symptoms rapidly progressed over days to include marked paraesthesia and weakness, particularly in the ulnar distribution of the left hand and the tibial distribution of the left lower limb. The asymmetry and rapid progression of these deficits were unusual for diabetic neuropathy, prompting further evaluation. Alongside the neurological decline, he experienced worsening fatigue, dysphagia, and unintentional weight loss. A gastroscopy was performed to evaluate for eosinophilic oesophagitis in the context of marked eosinophilia, but the findings were unremarkable.

**Figure 1:**
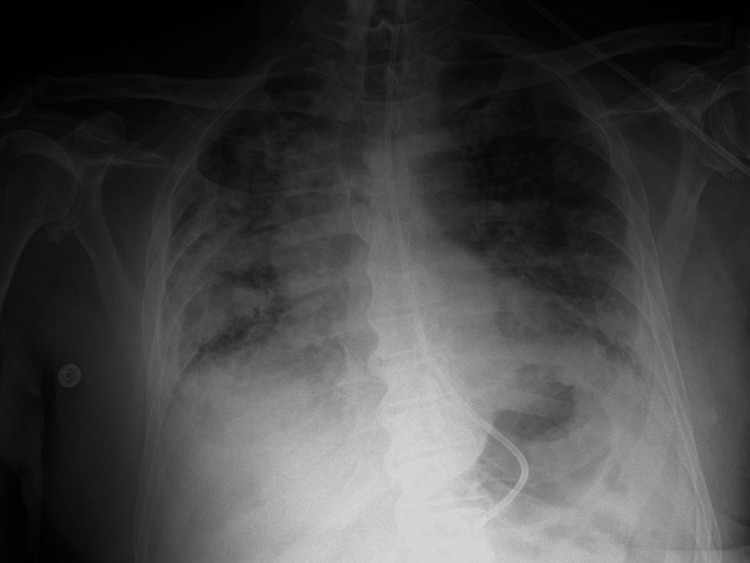
Chest radiograph demonstrating bilateral pulmonary opacities consistent with pulmonary haemorrhage, more pronounced in the right side. This image shows diffuse patchy opacities, greatest in the right mid-to-lower zones, consistent with vasculitic pulmonary involvement in eosinophilic granulomatosis with polyangiitis.

**Figure 2:**
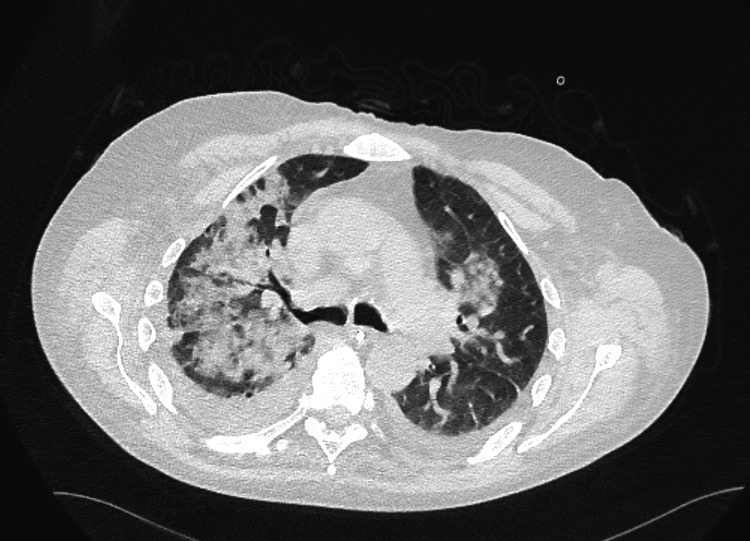
Computed tomography of the chest showing bilateral pulmonary haemorrhages, more pronounced in the right lung. Bilateral high-attenuation ground-glass and patchy consolidation, more pronounced on the right, suggest acute alveolar haemorrhage from a vasculitic process.

His systemic symptoms worsened with the onset of acute dyspnoea and haemoptysis, necessitating high-flow oxygen support. Imaging studies, including chest radiography (Figure 1) and subsequent computed tomography (Figure 2), revealed bilateral pulmonary haemorrhages, predominantly affecting the right lung, suggestive of an underlying vasculitic process. Differential diagnoses, including tuberculosis, were considered but subsequently excluded based on negative sputum analysis and interferon-gamma release assay (IGRA) results.

Laboratory investigations, as summarized in Table 1, revealed marked leukocytosis (WBC = 32 × 10⁹/L) with neutrophilia and eosinophilia (2.08 × 10⁹/L), along with an elevated C-reactive protein level (149 mg/L). MPO-ANCA was strongly positive (>134 U/mL), supporting the diagnosis of EGPA.

**Table 1:** Summary of key laboratory investigations relevant to the diagnosis of EGPA. Elevated eosinophils and strongly positive MPO-ANCA support a vasculitic process rather than an infectious aetiology. EGPA = eosinophilic granulomatosis with polyangiitis; MPO-ANCA = myeloperoxidase antineutrophil cytoplasmic antibody; TB = tuberculosis.

Laboratory tests	Results	Interpretation
White blood cell (WBC) count	32 * 10^9/L	Leukocytosis
Neutrophils	Increased	Neutrophilia
Eosinophils	2.08 * 10^9/L	Eosinophilia
C-reactive protein (CRP)	149 mg/L	Elevated
MPO-ANCA	>134 U/mL	Strongly positive (supporting the diagnosis of EGPA)
Sputum test for tuberculosis	Negative	No TB detected
Interferon-gamma release assay (IGRA)	Negative	No latent TB infection

As described above, the patient’s evolving neurological deficits, including asymmetric numbness and weakness, prompted further evaluation with electrophysiological studies. Nerve conduction studies (Tables 2-4) and electromyography (Table 5) were performed to characterize the pattern and extent of neuropathy. These investigations revealed asymmetric sensory and motor involvement consistent with mononeuritis multiplex. Specifically, there was impaired motor and sensory conduction in the right median and ulnar nerves, absent sural sensory responses bilaterally, and markedly reduced ulnar sensory responses with relative preservation of radial and lateral antebrachial cutaneous nerves. Motor studies demonstrated reduced compound muscle action potentials in intrinsic hand and foot muscles and slowed conduction in the right ulnar nerve.

**Table 2:** Sensory nerve conduction study findings demonstrating asymmetric sensory involvement. Onset Lat ms = onset latency (milliseconds); Peak Lat ms = peak latency (milliseconds); P-P Amp µV = peak-to-peak amplitude (microvolts); Dist. mm = distance (millimetres); Vel m/s = conduction velocity (meters per second); NR = no response; Rec = recording; Stim = stimulation; L = left; R = right.

Sensory nerve conduction					
Nerve/sites	Onset Lat ms	Peak Lat ms	P-P Lat ms	Dist. mm	Vel m/s
R Median - Ulnar Hand Sensory					
Digit 2 - Wrist	3.6	4.3	6.1	140	38.4
Digit 3 - Wrist	3.9	4.5	6.5	135	34.7
Digit 5 - Wrist	NR	NR	NR		
L Median - Ulnar Hand Sensory					
Digit 2 - Wrist	2.8	3.4	6.3	137	48.7
Digit 3 - Wrist	3	3.4	4.9	140	47.3
Digit 5 - Wrist	2.3	2.9	2.5	112	48
R Radial - Rec 1st Web Space, Stim Forearm					
Forearm	2.1	2.7	17.6	115	54.1
L Radial - Rec 1st Web Space, Stim Forearm					
Forearm	2.3	2.7	29.1	110	48.9
R Lateral Antebrachial Cutaneous - Rec Forearm Stim Elbow					
Elbow	1.3	1.9	8.5	90	69.7
L Lateral Antebrachial Cutaneous - Rec Forearm Stim Elbow					
Elbow	1.5	2	15.2	93	62
R Mixed Ulnar - Rec Elbow Stim Wrist					
Wrist	NR	NR	NR		
L Mixed Ulnar - Rec Elbow Stim Wrist					
Wrist	NR	NR	NR		
R Sural - Rec Ankle, Stim Lower Leg					
Lower Leg	NR	NR	NR		
L Sural - Rec Ankle, Stim Lower Leg					
Lower Leg	NR	NR	NR		

**Table 3:** Motor nerve conduction study results highlighting asymmetric axonal motor neuropathy. Onset ms = onset latency; P–P Amp mV = peak-to-peak amplitude; Lat Diff. ms = latency difference; Dist. mm = distance; Vel m/s = conduction velocity; NR = no response; Rec = recording; Stim = stimulation; ADM = abductor digiti minimi; APB = abductor pollicis brevis; EDB = extensor digitorum brevis; AH = abductor hallucis; L = left; R = right.

Motor nerve conduction						
Nerve/sites	Onset ms	P-P Amp mV	Segments	Lat Diff. ms	Dist. mm	Vel m/s
R Median - Rec APB Stim Wrist, Elbow, Axilla						
Wrist	NR	NR	Wrist - Elbow	NR		
R Median - Rec APB Stim Wrist, Elbow, Axilla						
Wrist	4.5	4.3	Wrist - Elbow	-6	275	45.8
Elbow	10.5	3.1	Elbow - Axilla			
R Ulnar - Rec ADM Stim Wrist, Elbow, Axilla						
Wrist	3	6.1	Wrist - Below Elbow	-5.5	230	42.1
Below Elbow	8.4	4.3	Below Elbow - Above Elbow	-2.1	116	54.6
Above Elbow	10.6	4.2	Above Elbow - Axilla			
L Median - Rec APB Stim Wrist, Elbow, Axilla						
Wrist	3.3	9.4	Wrist - Elbow	-5.5	270	48.9
Elbow	8.8	8.3	Elbow - Axilla			
L Ulnar - Rec ADM Stim Wrist, Elbow, Axilla						
Wrist	2.8	7.2	Wrist - Below Elbow	-4.6	230	50.4
Below Elbow	7.3	6.5	Below Elbow - Above Elbow	-1.6	90	55.4
Above Elbow	9	6.2	Above Elbow - Axilla			
R Peroneal - Rec EDB, Stim Ankle, Fibula, Pop Fossa						
Ankle	NR	NR	Ankle - Neck of Fibula	NR		
L Peroneal - Rec EDB, Stim Ankle, Fibula, Pop Fossa						
Ankle	NR	NR	Ankle - Neck of Fibula	NR		
R Tibial - Rec AH, Stim Ankle, Knee						
Ankle	NR	NR	Ankle - Knee	NR		
L Tibial - Rec AH, Stim Ankle, Knee						
Ankle	NR	NR	Ankle - Knee	NR		

**Table 4:** F-wave studies demonstrating prolonged latencies consistent with proximal nerve involvement. M Lat ms = distal motor latency in milliseconds; F Lat ms = F-wave latency in milliseconds; APB = abductor pollicis brevis; ADM = abductor digiti minimi; L = left; Nerve = stimulated peripheral nerve; ms = milliseconds.

F-wave studies		
Nerve	M Lat ms	F Lat ms
L Median - APB	3.4	32.6
L Ulnar - ADM	2.9	31.8

**Table 5:** Electromyography (EMG) summary demonstrating active and chronic denervation consistent with vasculitic mononeuritis multiplex. Ins. Activity = insertional activity; Spont. Activity = spontaneous activity; Fib = fibrillation potentials; + Wave = positive sharp waves; Fasc = fasciculations; Volitional MUAPs = volitional motor unit action potentials; Dur. = duration; Amp = amplitude; Poly = polyphasic potentials; Recruit = recruitment; Max. Volitional Act. = maximal volitional activation.

Electromyography summary										
	Ins. Activity	Spont. Activity			Volitional MUAPs				Maz. Volitional Act.	
Muscle	Insertional	Fib	+ Wave	Fasc	Dur.	Amp	Poly	Recruit	Pattern	Effort
R. Tibialis Anterior	Myotonia	1+	1+	None	Normal	Low	None	Reduced	Single Unit	Max
L. Tibialis Anterior	Myotonia	1+	2+	None					None	Max
R. Gastrocnemius (Medial Head)	Myotonia	1+	2+	None	Normal	Normal	None	Reduced	Discrete	Max
L. Gastrocnemius (Medial Head)	Myotonia	1+	1+	None	Normal	Normal	None	Normal	Discrete	Max
R. Vastus Medialis	Normal	None	None	None	Long	High	None	Reduced	Discrete	Max
L. Vastus Medialis	Normal	None	None	None	Long	High	None	Reduced	Discrete	Max
R. Deltoid	Normal	None	None	None	Normal	Normal	Many	Normal	Moderate	Max
R. Extensor Digitorum Communis	Normal	None	None	None	Normal	Normal	None	Normal	Full	Max
R. First Dorsal Interosseous	Normal	None	None	None	Long	Normal	None	Reduced	Discrete	Max
L. First Dorsal Interosseous	Normal	None	None	None	Normal	Normal	Many	Reduced	Moderate/Discrete	Max
R. Iliopsoas	Normal	None	None	None	Normal	Normal	None	Normal	Full	Max
R. T10 Paraspinal	Normal	None	None	None						
R. T8 Paraspinal	Increased	1+	1+	None						
L. T8 Paraspinal	Increased	None	None	None						
R. T7 Paraspinal	Increased	1+	1+	None	Normal	Normal	None	Normal	Full	Max

Electromyography (EMG) demonstrated fibrillation potentials and positive sharp waves in the tibialis anterior, medial gastrocnemius, and thoracic paraspinal muscles, chronic reinnervation changes in the vastus medialis and first dorsal interosseous muscles, and fibrosis in the medial gastrocnemius. Overall, the findings indicate an asymmetric, predominantly axonal, inflammatory neuropathy with both chronic and active denervation, consistent with mononeuritis multiplex.

A multidisciplinary discussion between neurology and rheumatology teams confirmed vasculitic mononeuritis multiplex secondary to EGPA. The patient was treated with high-dose intravenous corticosteroids, pulsed methylprednisolone, and cyclophosphamide. He later developed sepsis, requiring intensive care support, including plasmapheresis, intravenous immunoglobulin, and mechanical ventilation.

Following stabilization, rituximab therapy was initiated. Over the following weeks, the patient showed marked improvement in systemic symptoms and neurological function, with gradual recovery of motor and sensory deficits.

## Discussion

This case underscores the diagnostic and therapeutic complexity of EGPA in a patient with pre-existing diabetes mellitus. While neuropathy is a common complication of diabetes, the presence of asymmetric or patchy deficits, particularly when accompanied by systemic inflammation, eosinophilia, or pulmonary involvement, should prompt consideration of a vasculitic aetiology such as EGPA [5-7]. Unlike diabetic polyneuropathy, which typically presents as a symmetric, distal sensorimotor neuropathy without systemic manifestations, vasculitic neuropathy often demonstrates asymmetry, rapid progression, and nerve-specific involvement, as observed in this patient [5].

Electrophysiological studies and serological testing, particularly MPO-ANCA, were pivotal in distinguishing EGPA-associated neuropathy from diabetic neuropathy [3,4,6]. In this patient, nerve conduction studies revealed asymmetric axonal involvement, with selective impairment of the ulnar, median, and tibial nerves, while other sensory nerves, such as the radial and lateral antebrachial cutaneous nerves, were relatively preserved. This pattern, coupled with EMG evidence of fibrillation potentials, positive sharp waves, and chronic reinnervation, indicated ongoing inflammatory damage and differentiated the neuropathy from the diffuse, length-dependent axonal loss typically seen in diabetes [4]. The strongly positive MPO-ANCA and marked eosinophilia further supported a systemic vasculitic process rather than metabolic neuropathy alone.

The patient’s clinical course also highlights the potential severity of EGPA, exemplified by pulmonary haemorrhage and sepsis requiring intensive care management [9]. The favourable response to immunosuppressive therapy, including corticosteroids, cyclophosphamide, and rituximab, demonstrates the critical importance of early recognition and aggressive intervention to prevent permanent neurological deficits and multi-organ complications [8,9].

Overall, this report contributes to the literature by reinforcing the need for vigilance in diabetic patients presenting with atypical, asymmetric neuropathies, especially when accompanied by systemic or inflammatory features [5,10]. The synthesis of electrophysiological findings with serological and clinical data illustrates how careful interpretation beyond descriptive reporting can guide early, targeted therapy, ultimately reducing morbidity and preventing irreversible organ damage [9].

## Conclusions

EGPA should be actively considered in diabetic patients who develop unexplained systemic symptoms and asymmetric neuropathy. Differentiating vasculitic neuropathy from diabetic neuropathy requires thorough clinical evaluation, electrophysiological studies, and serological testing for ANCA and eosinophil counts.

Prompt recognition and initiation of immunosuppressive therapy, supported by a multidisciplinary care approach, are crucial to prevent permanent neurological deficits and organ damage. This case highlights the importance of maintaining a high index of suspicion for EGPA in complex diabetic presentations to ensure timely diagnosis and improved patient outcomes.

## References

[1] Fijolek J, Radzikowska E (2023). Eosinophilic granulomatosis with polyangiitis - advances in pathogenesis, diagnosis, and treatment. Front Med (Lausanne).

[2] Gioffredi A, Maritati F, Oliva E, Buzio C (2014). Eosinophilic granulomatosis with polyangiitis: an overview. Front Immunol.

[3] Trivioli G, Terrier B, Vaglio A (2020). Eosinophilic granulomatosis with polyangiitis: understanding the disease and its management. Rheumatology (Oxford).

[4] Cho HJ, Yune S, Seok JM (2017). Clinical characteristics and treatment response of peripheral neuropathy in the presence of eosinophilic granulomatosis with polyangiitis (Churg-Strauss syndrome): experience at a single tertiary center. J Clin Neurol.

[5] Kheng QK (2017). Eosinophilic granulomatosis with polyangiitis (EGPA)- a presentation of rapid progressive demyelinating polyneuropathy. Open J Clin Med Case Rep.

[6] Kalinova D, Kukushev G, Kolarov Z, Rashkov R (2019). Severe mononeuritis multiplex in a patient with eosinophilic granulomatosis with polyangiitis. Reumatologia.

[7] Shah T, Patel P, Patel M (2025). Unique case of mononeuritis multiplex in a patient of Eosinophilic granulomatosis with polyangiitis. J Family Med Prim Care.

[8] Fanouriakis A, Kougkas N, Vassilopoulos D, Fragouli E, Repa A, Sidiropoulos P (2015). Rituximab for eosinophilic granulomatosis with polyangiitis with severe vasculitic neuropathy: case report and review of current clinical evidence. Semin Arthritis Rheum.

[9] Baikunje S, Vankalakunti M, Upadhyaya VS, Hosmane GB (2016). Eosinophilic granulomatosis with polyangiitis with severe pulmonary hemorrhage treated with rituximab. Indian J Nephrol.

[10] England JD, Asbury AK (2004). Peripheral neuropathy. Lancet.

